# Proposed revision to the taxonomy of the genus *Pestivirus,* family *Flaviviridae*

**DOI:** 10.1099/jgv.0.000873

**Published:** 2017-08-08

**Authors:** Donald B. Smith, Gregor Meyers, Jens Bukh, Ernest A. Gould, Thomas Monath, A. Scott Muerhoff, Alexander Pletnev, Rebecca Rico-Hesse, Jack T. Stapleton, Peter Simmonds, Paul Becher

**Affiliations:** ^1^​ Centre for Immunity, Infection and Evolution, University of Edinburgh, Scotland, UK; ^2^​ Nuffield Department of Medicine, University of Oxford, UK; ^3^​ Institut für Immunologie, Friedrich-Loeffler-Institut, Federal Research Institute for Animal Health, Greifswald-Insel Riems, Germany; ^4^​ Copenhagen Hepatitis C Program (CO-HEP), Department of Infectious Diseases and Clinical Research Centre, Copenhagen University Hospital, Hvidovre, and Department of Immunology and Microbiology, Faculty of Health and Medical Sciences, University of Copenhagen, Denmark; ^5^​ Aix Marseille Université, IRD French Institute of Research for Development, EHESP French School of Public Health, EPV UMR_D 190 Emergence des Pathologies Virales, Marseille, France; ^6^​ NewLink Genetics Corp, Infectious Diseases Division, Devens MA, USA; ^7^​ Abbott Diagnostics Research and Development, Abbott Park, IL, USA; ^8^​ Laboratory of Infectious Diseases, National Institute of Allergy and Infectious Diseases (NIAID), National Institutes of Health (NIH), Bethesda, MD, USA; ^9^​ Molecular Virology & Microbiology and Department of Pediatrics, Baylor College of Medicine, Houston, TX, USA; ^10^​ Medical Service, Iowa City Veterans Affairs Medical Center, Departments of Internal Medicine and Microbiology, University of Iowa, Iowa City, IA, USA; ^11^​ Institute of Virology, University of Veterinary Medicine, Hannover, Germany

**Keywords:** *Pestivirus*, taxonomy, ICTV

## Abstract

We propose the creation of seven new species in the genus *Pestivirus* (family *Flaviviridae*) in addition to the four existing species, and naming species in a host-independent manner using the format *Pestivirus X*. Only the virus species names would change; virus isolates would still be referred to by their original names. The original species would be re-designated as *Pestivirus A* (original designation *Bovine viral diarrhea virus 1*), *Pestivirus B* (*Bovine viral diarrhea virus 2*), *Pestivirus C* (*Classical swine fever virus*) and *Pestivirus D* (*Border disease virus*). The seven new species (and example isolates) would be *Pestivirus E* (pronghorn pestivirus), *Pestivirus F* (Bungowannah virus), *Pestivirus G* (giraffe pestivirus), *Pestivirus H* (Hobi-like pestivirus), *Pestivirus I* (Aydin-like pestivirus), *Pestivirus J* (rat pestivirus) and *Pestivirus K* (atypical porcine pestivirus). A bat-derived virus and pestiviruses identified from sheep and goat (Tunisian sheep pestiviruses), which lack complete coding region sequences, may represent two additional species.

## Abbreviations

APPV, atypical porcine pestivirus; BDV, Border disease virus; BVDV-1, Bovine viral diarrhea virus 1; BVDV-2, Bovine viral diarrhea virus 2; CSFV, Classical swine fever virus; cp, cytopathogenic; ICTV, International Committee on Taxonomy of Viruses.

## Full-Text

The genus *Pestivirus* in the family *Flaviviridae* currently comprises four species, *Bovine viral diarrhea virus 1* (BVDV-1), *Bovine viral diarrhea virus 2* (BVDV-2), *Border disease virus* (BDV) and *Classical swine fever virus* (CSFV) [1, 2]. Pestiviruses infect pigs and ruminants with significant economic impact [3, 4], but have also been detected in wild ruminants and wild boar [5, 6]. Pestiviruses have a positive-sense single-stranded RNA genome that encodes a single polyprotein cleaved co- and post-translationally by cellular and viral proteases into four structural and eight non-structural proteins. Translation of genomic RNA is initiated internally by a cap-independent mechanism through a type IV internal ribosomal entry site within the 5′-non-coding region of the virus genomic RNA [7].

Proteins unique to the *Pestivirus* genus are the E^rns^ envelope glycoprotein, which has RNase activity, and the non-structural protease N^pro^, which releases itself auto-catalytically from the polyprotein. Both proteins are implicated in blocking the host antiviral defence [7]. Evolution of pestiviruses occurs by point mutation and by homologous recombination within species [8, 9]. In addition, while most pestiviruses are non-cytopathogenic (non-cp), both non-cp and cytopathogenic (cp) virus variants have been described for all four currently classified species. Cp variants typically arise by various non-homologous recombination events, including the insertion of host-derived protein-coding RNA sequences. Alternatively, introduction of sets of mutations within NS2 can lead to cp viruses [10]. In any case, the genome alterations in cp viruses result in unlimited production of NS3 during the virus replication cycle [7, 9].

The four existing *Pestivirus* species are demarcated using a range of criteria including complete coding nucleotide sequences that differ by more than 25 %, displaying >10-fold differences in cross-neutralization titres, and may have differing, although overlapping, host ranges [1, 2]. However, several publications have described additional viruses related to pestiviruses, but which are genetically distinct and may represent additional species within the genus. These viruses have been isolated from domestic animals [11–16] and wild species such as giraffes [5, 17] and pronghorn antelopes [18, 19]. Recently, pestivirus sequences have been detected in rats [20] and bats [21], although virus isolates have not been obtained. While these and other studies [2, 6, 22, 23] have proposed the existence of additional pestivirus species, there has been no change to the taxonomy of the genus since 1999.

This paper describes proposals from the *Flaviviridae* Study Group of the International Committee for the Taxonomy of Viruses (ICTV) to revise the taxonomy of the genus *Pestivirus* to include seven additional species, to name all *Pestivirus* species according to a standard format and to modify the criteria by which pestivirus species are demarcated. Similar analytical methods and taxonomic naming schemes have recently been proposed by the same group for a revised taxonomy of the *Hepacivirus* and *Pegivirus* genera in this virus family [24].

## Diversity between members of existing *Pestivirus* species

Pestivirus sequences were downloaded from GenBank on 22 February 2017 using the search term ‘Pestivirus 5000[SLEN]:20000[SLEN]’. Complete coding region sequences were aligned within the SSE package v1.2 [25] using muscle [26] followed by manual editing to remove host-derived insertions and duplicate sequences. The final alignment of 320 sequences comprised BVDV-1 (*n*=85), BVDV-2 (99), BDV [11], CSFV (99) and others [26].

Pairwise nucleotide and amino acid p-distances (the proportion of non-identical sites) between complete coding sequences of members of the four existing species in the *Pestivirus* genus were <0.3 and <0.27 (BVDV-1), <0.17 and <0.11 (BVDV-2), <0.24 and <0.15 (BDV) and <0.19 and <0.13 (CSFV). The greater range of distances observed for BVDV-1 was due to comparisons involving the sequences JQ799141, U86599 and U86600. The first of these, derived from a yak, contains a double frameshift that results in a cluster of 21 amino acid substitutions, as well as 16 in-frame termination codons clustered in the 3′ half of the genome and five 1 nt deletions that would disrupt translation of the polyprotein. The latter two sequences are closely related to each other and contain a region with a cluster of 31 amino acid substitutions in a 36 amino acid region produced by three frameshift mutations, as well as a 38 amino acid mismatched stretch of uncertain origin. When these three sequences were excluded from comparisons, nucleotide and amino acid p-distances were <0.23 and <0.15, similar to the range for the other three species.

In contrast, inter-species nucleotide and amino acid p-distances were all outside this range at >0.28 and >0.2, respectively, mirroring patterns of virus host range and cross-neutralization, and together supporting the existing taxonomy where BVDV-1, BVDV-2, CSFV and BDV belong to four different species [2, 5] (Table 1).

**Table 1.** Characteristics of proposed pestivirus species

Existing species name	Proposed species name	Virus names	Abbreviation	Isolate type	GenBank Accession	Host	Complete coding region sequences	Disease
*Bovine viral diarrhea virus 1*	*Pestivirus A*	bovine viral diarrhea virus 1	BVDV-1	NADL	M31182	Cattle, sheep, other ruminants, pig	79	Bovine viral diarrhea/ mucosal disease (BVD/MD)
*Bovine viral diarrhea virus 2*	*Pestivirus B*	bovine viral diarrhea virus 2	BVDV-2	890	U18059	Cattle, sheep, other ruminants pig	99	BVD/MD
*Classical swine fever virus*	*Pestivirus C*	classical swine fever virus, hog cholera virus	CSFV	A187	X87939	Pig	96	Classical swine fever
*Border disease virus*	*Pestivirus D*	Border disease virus, reindeer pestivirus	BDV	X818	AF037405	Sheep, reindeer, chamois, other ruminants, pig	13	Border disease Hairy shaker syndrome Fuzzy lamb syndrome
	*Pestivirus E*	pronghorn antelope pestivirus	Pronghorn		AY781152	Antelope	1	Unknown
	*Pestivirus F*	Bungowannah virus	Bungo	Bungowannah	EF100713	Pig	1	Porcine myocarditis syndrome
	*Pestivirus G*	giraffe pestivirus	Giraffe	H138	AF144617	Giraffe, cattle	2	MD-like (giraffe)/unknown (cattle)
	*Pestivirus H*	Hobi-like pestivirus, atypical ruminant pestivirus, bovine viral diarrhea virus 3	Hobi-like, BVDV-3	Th/04_KhonKaen	FJ040215	Cattle, buffalo	12	BVD/MD
	*Pestivirus I*	Aydin-like pestivirus,		Aydin/04-TR	JX428945	Sheep, goat	2	Abortions, congenital malformations
	*Pestivirus J*	rat pestivirus		NrPV/NYC-D23	KJ950914	Rat	1	Unknown
	*Pestivirus K*	atypical porcine pestivirus	APPV	000515	KR011347	Pig	6	Congenital tremor

## Unassigned *Pestivirus* sequences

Using the maximum nucleotide and amino acid distances described above for members of the four existing *Pestivirus* species as demarcation criteria, we next analysed the relationship between these species and currently unassigned isolates. The complete coding sequence of the reindeer pestivirus (AF144618, V60-Krefeld [17]) showed nucleotide and amino acid p-distances of <0.24 and <0.15 in comparisons with members of the species *Border disease virus* and so can be considered as a member of that species. This conclusion is supported by previous studies of sequence relationships among pestiviruses and antigenic relatedness determined by cross-neutralization that concluded that the reindeer pestivirus together with related ovine viruses belongs to a separate genotype within the species *Border disease virus* [2].

In contrast, nucleotide and amino acid p-distances exceeded 0.28 and 0.19, respectively, for comparisons between giraffe pestivirus (AF144617), pronghorn antelope pestivirus (AY781152), Bungowannah virus (EF100713), Hobi-like pestivirus (FJ040215), rat pestivirus (KJ950914), atypical porcine pestivirus (KR011347) and Aydin-like pestivirus (JX428945) in comparisons with each other and members of the four existing *Pestivirus* species (Table S1, available in the online Supplementary Material).

**Fig. 1.**
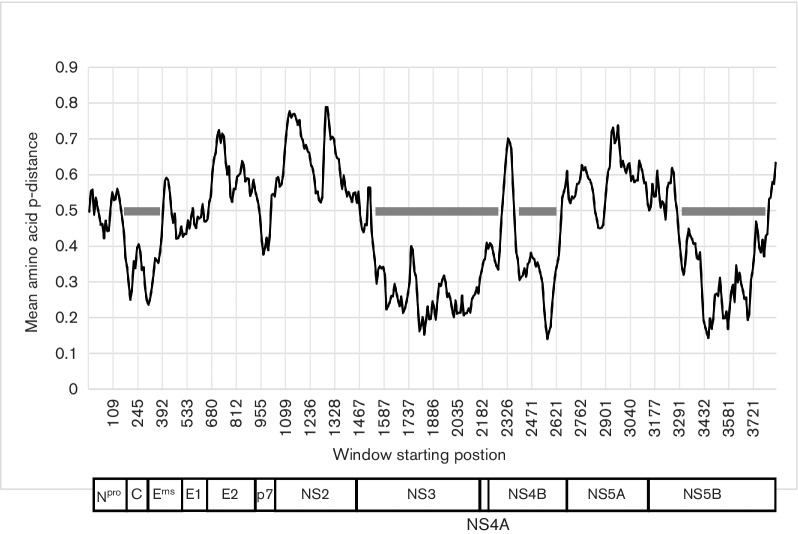
Amino acid sequence diversity across pestivirus genomes. Mean amino acid p-distances were computed for a sliding window of 50 amino acids shifted by 10 residues across the complete coding region for comparisons between single representatives of each accepted and proposed *Pestivirus* species. Positions are numbered relative to the polyprotein of BVDV-1 SD-1 (M96751). Intervals on the x-axis are not regular because of un-numbered alignment gaps. Four regions where mean distances are consistently <0.5 are indicated by grey bars. A schema of the proteins encoded by the virus genome is provided below.

**Fig. 2.**
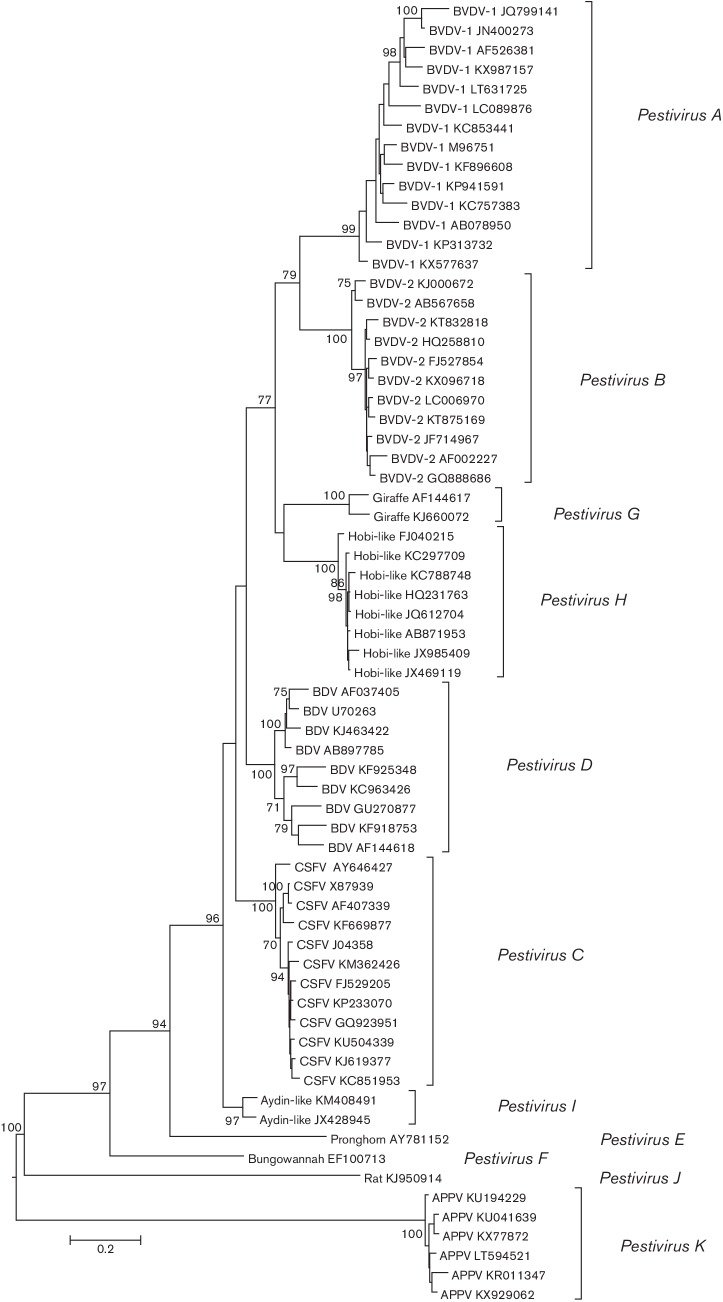
Phylogenetic analysis of pestivirus polyprotein fragments. Phylogenetic trees were constructed using mega 6 [36] and based upon distances between amino acid sequences for amino acid positions 3312–3899 by maximum likelihood using a JTT+G model. Up to 15 sequences were used for each species, choosing the most divergent sequences and eliminating sequences <1 % divergent, and comprised: BVDV1 (M96751, JQ799141, KP313732, KP941591, JN400273, KF896608, KC757383, KC853441, AB078950, AF526381, LC089876, KX577637, KX987157, LT631725), BVDV2 (AF002227, LC006970, KT875169, KT832818, KJ000672, HQ258810, JF714967, AB567658, FJ527854, GQ888686, KX096718), CSFV (X87939, J04358, FJ529205, AY646427, KF669877, KP233070, KM362426, KJ619377, KC851953, GQ923951, AF407339, KU504339), BDV (AF037405, AB897785, KJ463422, KF925348, KF918753, KC963426, GU270877, U70263, AF144618), Hobi-like (FJ040215, KC788748, KC297709, JX985409, JX469119, JQ612704, HQ231763, AB871953), giraffe (AF144617, KJ660072), Aydin-like (KM408491, JX428945), pronghorn (AY781152), rat (KJ950914), Bungowannah (EF100713), APPV (KU041639, KR011347, KU194229, LT594521, KX77872, KX929062). Branches supported by >70 % of bootstrap replicates are indicated.

A scan of amino acid variability across pestivirus genomes (50 residue window shifted by 10 residues) using a single representative from each existing or proposed species (BVDV-1/M96751, BVDV-2/AF002227, CSFV/AF326963, BDV/AF037405, Hobi-like/FJ040215, giraffe/AF144617, pronghorn/AY781152, Bungowannah/EF100713, Aydin-like/JX428945, rat/KJ950914, APPV/KU041639) revealed several regions of sequence conservation with mean p-distances consistently <0.5 and therefore potentially more suitable for phylogenetic analysis (Fig. 1). These conserved regions correspond to amino acid positions 189–418, 1547–2321, 2397–2688 and 3312–3837 (numbered according to the first amino acid of the polyprotein of BVDV-1 SD-1, accession number M96751). Phylogenetic analysis of amino acid sequences of a reduced set of isolates in the region 3312–3837 (corresponding to most of the NS5B protein) provided support for 11 groups of sequences (Fig. 2). Members of the four existing species grouped more closely with giraffe pestivirus, Hobi-like pestivirus and Aydin-like pestivirus sequences, while much longer branches were observed between them and the antelope pestivirus, Bungowannah virus, atypical porcine pestivirus and rat pestivirus sequences. Sequences from each species formed a distinct clade supported by >70 % of bootstrap replicates. Similar results were obtained from phylogenetic analysis of the three other conserved subgenomic regions (Fig. S1). These analyses support the assignment of pestiviruses for which complete genome sequences are available in 11 species.

A single incomplete genome sequence of a pestivirus obtained from a bat is also available (JQ814854) and differs considerably from all other known members of existing and proposed species, with amino acid p-distances of >0.33 over 1710 residues (positions 629–2610, numbered as above). Comparisons over the same region between the four existing species or the 11 proposed species gave amino acid p-distances of 0.18 to 0.29 and 0.18–0.67 respectively, suggesting that this virus also may belong to an additional species. Similarly, phylogenetic analysis for the regions 1547–2321 and 2397–2607 showed that the bat pestivirus sequence grouped with atypical porcine pestivirus (APPV), but with a branch equal to or deeper than that observed between existing or proposed pestivirus species (data not shown). Additional phylogenetic groupings have been reported based on the analysis of subgenomic regions of viruses isolated from sheep and goats [27–29]. The future description and analysis of complete coding sequences will likely confirm their membership of additional species.

## Biological characteristics assisting in *Pestivirus* species demarcation

These proposed species assignments are consistent with previously reported structural and biological characteristics. For example, members of the different *Pestivirus* species can be distinguished from each other by the presence of sequence motifs in the 5′-untranslated region that are involved in RNA secondary structures [30]. Similarly, antigenic relationships have been studied for the four established and some of the proposed pestivirus species by cross-neutralization studies, with clear antigenic differences between members of different species [2, 12, 17, 31, 32] although BVDV-1 cross-protects against BVDV-2 in challenge studies [33]. However, antigenic relationships have not been investigated for the rat pestiviruses since no virus isolates are available and so its proposed classification is exclusively based on sequence analysis and its presumed host.

There are less clear-cut differences in host range (Table 1). BVDV-1, BVDV-2 and BDV can infect a wide range of ruminants, including cattle, sheep, goats, and a number of wild ruminants as well as pigs. Moreover, for several of the newly proposed species the full extent of host range has yet to be discovered.

## Naming of *Pestivirus* species

The current *Pestivirus* species names are derived from virus isolate names, and these in turn are based on various host range and disease attributes. For example, the species name *Bovine viral diarrhea virus 1* describes the host species of the first isolate and aspects of the disease it causes, followed by a number to distinguish it from *Bovine viral diarrhea virus 2*. A similar format is used for the species *Classical swine fever virus* (previously called *Hog cholera virus*), but the species name *Border disease virus* comes from the geographical location of the first isolates (the border between England and Wales in the UK). Other unclassified pestivirus isolates have been named after their host (giraffe pestivirus, pronghorn antelope pestivirus, rat pestivirus, atypical porcine pestivirus) or the geographical location of the first isolate (Bungowannah virus, Aydin-like pestivirus).

One problem with this naming system is that there is no clear distinction between the taxonomic category (species name) and the physical agent (a virus) apart from the italicization and presence of an initial capital of the former. For example, the species *Border disease virus* includes the virus Border disease virus from sheep and goat (with an initial capital in this case because ‘Border’ is a proper noun), but also reindeer pestivirus and further variants infecting pigs, cattle and bison. In addition, with the exception of *Classical swine fever* virus, members of which under natural conditions exclusively infect domestic pigs and wild boar, the current descriptive species names are misleading with respect to host range (Table 1). Finally, the current species names give no hint as to the virological and pathological similarities between different members of the genus *Pestivirus.*

We propose a new uniform naming system for species with the format *Pestivirus X*, where *X* represents a different capital letter for each species (Table 1). The four existing species become *Pestivirus A* (*Bovine viral diarrhea virus 1), Pestivirus B* (*Bovine viral diarrhea virus 2), Pestivirus C* (*Classical swine fever virus)* and *Pestivirus D* (*Border disease virus)*. As described, this proposal only relates to the nomenclature of these species; the naming of the virus isolates or variants would not change, so for example the NADL isolate could still be described as bovine viral diarrhea virus 1 NADL, but would become a member of the species *Pestivirus A*. This format mirrors that used for species belonging to the *Flaviviridae* genera *Pegivirus* [34] and *Hepacivirus* [24]. We have assigned species names to the seven additional pestivirus species in alphabetical order roughly in the chronological order of their discovery, but with deviations to accommodate memorable pairings such as *Pestivirus G* with giraffe pestivirus (Table 1).

A consequence of our proposed classification is that some of the proposed species are largely based on sequence relationships between viruses for which disease associations or veterinary consequences are largely unknown. Future work describing the virome of a wider range of host species is likely to identify many additional pestiviruses for which pathological information may again be lacking. In contrast, all four current *Pestivirus* species include viruses that are important veterinary pathogens, and it could be argued that there is little utility in establishing additional pestivirus species in the absence of information about their biology and pathogenicity. Our decision to only create new species when a virus complete coding region sequence was available has the effect of limiting the proliferation of species names to those viruses that have been of sufficient interest to merit at least this investment of time and effort. The creation of a robust, sequence-based classification of pestiviruses will be of considerable value for classifying members of this genus in the future.

Our taxonomic revision of the genus *Pestivirus* into 11 species does not accurately reflect the hierarchy of sequence relationships observed within the genus; members of the species *Pestivirus J* and *Pestivirus K* were more divergent from members of other species and each other (amino acid p-distances over complete polyprotein of 0.58–0.67) than members of the species *Pestivirus A, Pestivirus B, Pestivirus C and Pestivirus D* were from each other (distances of 0.18–0.29) or than for members of existing and proposed pestivirus species excluding *Pestivirus J* and *Pestivirus K* (0.19–0.48). The current ICTV classification framework does not include a sub-genus category, but nevertheless we do not support the division of the *Pestivirus* genus into multiple genera; all 11 proposed species share common genome organization, protein homology and, where known, virological features and pathogenicity. In addition, equivalent diversity is observed for other genera within the *Flaviviridae*. Amino acid p-distances for a region of NS5B (positions 3453–3749, numbered as above, using the alignment available at www.ictv.global/report/flaviviridae) were 0.23–0.63 between members of 14 *Hepacivirus* species, 0.23–0.59 between members of 11 *Pegivirus* species, and 0.02–0.44 between members of 53 *Flavivirus* species; this compares with 0.07–0.38 between members of the 11 proposed *Pestivirus* species and >0.58 for members of different genera. The pestivirus sequences were monophyletic in a phylogenetic tree constructed using these NS5B amino acid sequences [1].

The ranges of inter-species distances observed within different genera within the *Flaviviridae* reflect the different weight given to biological, serological and geographical characters in assigning viruses to different species. In the case of pestiviruses, the assignment of species reflects the originally described differences in host range and pathogenicity of the first variants to be characterized. The species proposals in the current study are consistent with and extend this initially utilitarian classification approach. Note added in proof: After acceptance of the manuscript an additional porcine pestivirus (Linda) was reported to be distinct from the eleven pestivirus species described here [35].

## Supplementary Data

326 Supplementary File 1
