# Supplementary material for: Proposed revision to the taxonomy of the genus Pestivirus, family Flaviviridae
Source: J Gen Virol. 2017 Aug 8;98(8):2106–12. doi: 10.1099/jgv.0.000873 (PMC5656787; doi:10.1099/jgv.0.000873)
Supplement: Supplementary File 1 [file jgv-98-2106-s001.pdf]

Supplementary Figure 1A

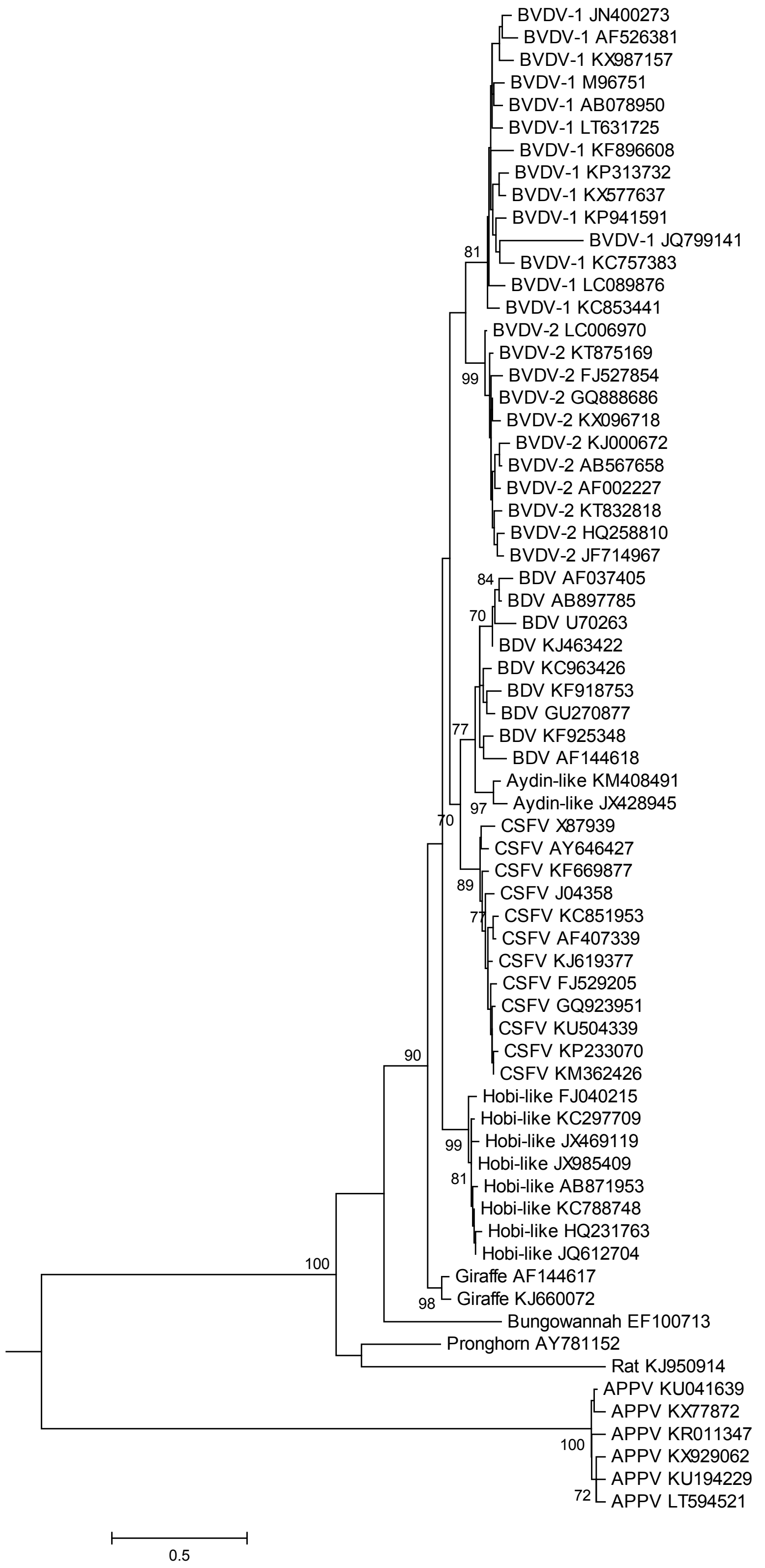

Supplementary Figure 1B

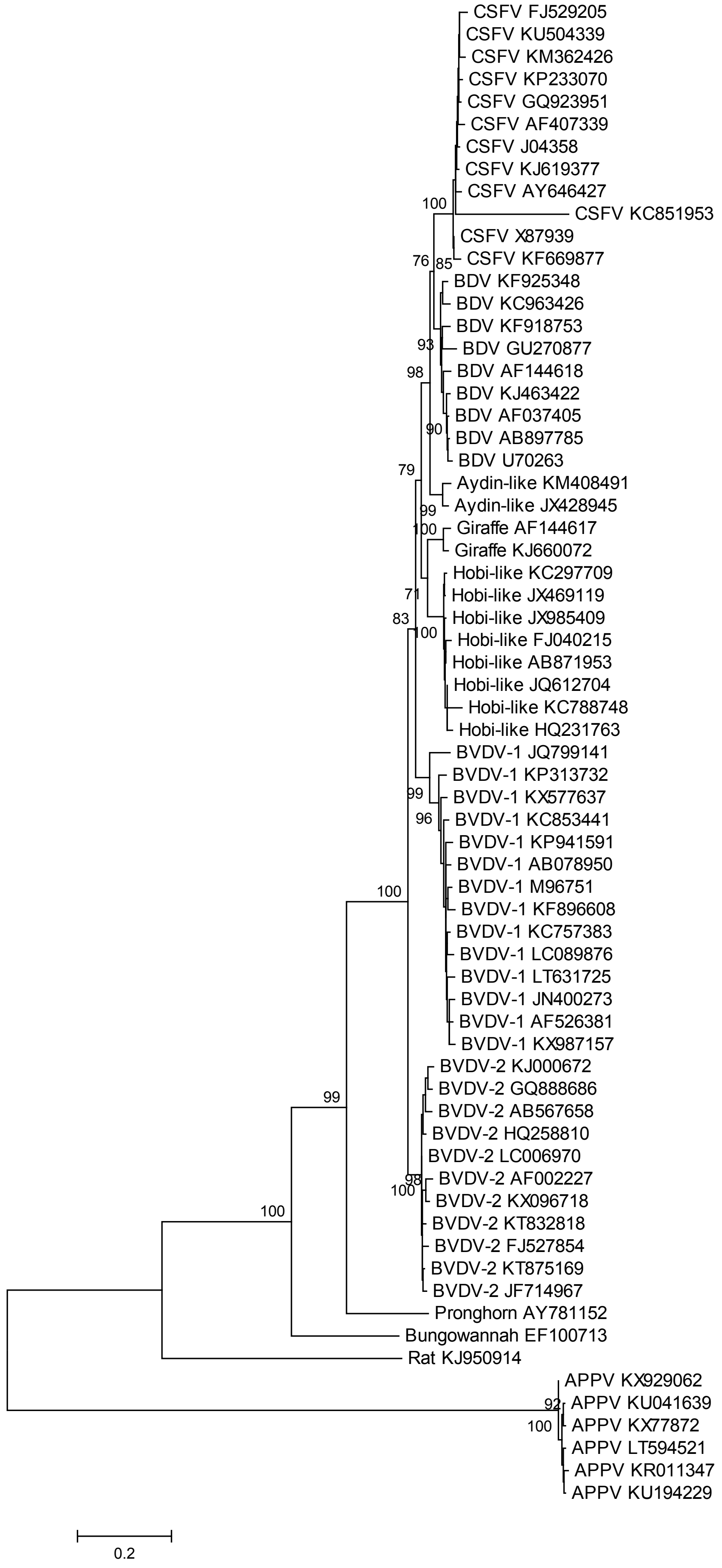

Supplementary Figure 1C

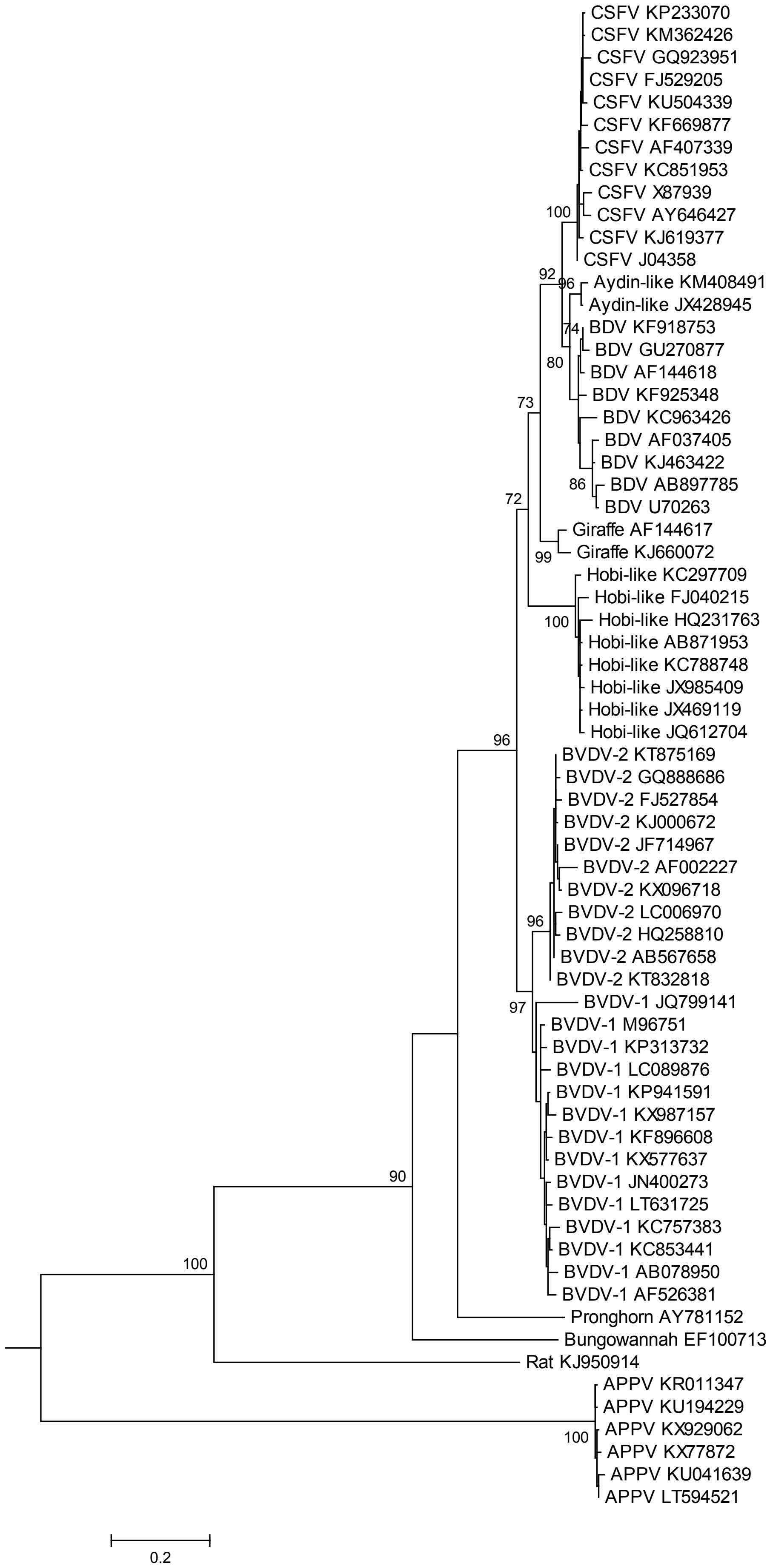

**Figure legend Supplementary Figure 1**

Phylogenetic analysis of pestivirus polyprotein fragments. Phylogenetic trees were constructed using MEGA 6 (35) and based upon distances between amino acid sequences for amino acid positions A. 189-418, B. 1547-2321 and C. 2397-2688 by maximum likelihood using a JTT+G model. Up to fifteen sequences were used for each species, choosing the most divergent sequences and eliminating sequences < 1% divergent, and comprised: BVDV1 (M96751, JQ799141, KP313732, KP941591, JN400273, KF896608, KC757383, KC853441, AB078950, AF526381, LC089876, KX577637, KX987157, LT631725), BVDV2 (AF002227, LC006970, KT875169, KT832818, KJ000672, HQ258810, JF714967, AB567658, FJ527854, GQ888686, KX096718), CSFV (X87939, J04358, FJ529205, AY646427, KF669877, KP233070, KM362426, KJ619377, KC851953, GQ923951, AF407339, KU504339), BDV (AF037405, AB897785, KJ463422, KF925348, KF918753, KC963426, GU270877, U70263, AF144618), Hobi-like (FJ040215, KC788748, KC297709, JX985409, JX469119, JQ612704, HQ231763, AB871953), Giraffe (AF144617, KJ660072), Aydin-like (KM408491, JX428945), Pronghorn (AY781152), Rat (KJ950914), Bungowannah (EF100713), APPV (KU041639, KR011347, KU194229, LT594521, KX77872, KX929062). Branches supported by >70% of bootstrap replicates are indicated.

**Supplementary Table 1** Nucleotide (upper section) and amino acid (lower section) p-distances between complete pestivirus coding sequences

| | BVDV-1 M96751 | BVDV-2 AF002227 | Hobi-like FJ040215 | Giraffe AF144617 | Pronghorn AY781152 | APPV KU041639 | BDV AF037405 | CSFV AF326963 | Aydin-like JX428945 | Rat KJ950914 | Bungowannah EF100713 |
|---|---|---|---|---|---|---|---|---|---|---|---|
| BVDV-1/M96751 | | 0.307 | 0.322 | 0.324 | 0.398 | 0.534 | 0.324 | 0.329 | 0.324 | 0.506 | 0.438 |
| BVDV-2/AF002227 | 0.252 | | 0.325 | 0.325 | 0.398 | 0.535 | 0.332 | 0.334 | 0.336 | 0.504 | 0.436 |
| Hobi-like/FJ040215 | 0.277 | 0.295 | | 0.323 | 0.396 | 0.535 | 0.327 | 0.325 | 0.322 | 0.506 | 0.430 |
| Giraffe/AF144617 | 0.277 | 0.291 | 0.274 | | 0.405 | 0.538 | 0.316 | 0.323 | 0.318 | 0.508 | 0.433 |
| Pronghorn/AY781152 | 0.409 | 0.414 | 0.405 | 0.412 | | 0.554 | 0.394 | 0.399 | 0.395 | 0.500 | 0.439 |
| APPV/KU041639 | 0.640 | 0.646 | 0.642 | 0.644 | 0.659 | | 0.537 | 0.538 | 0.539 | 0.553 | 0.551 |
| BDV/AF037405 | 0.278 | 0.292 | 0.277 | 0.268 | 0.410 | 0.644 | | 0.283 | 0.282 | 0.502 | 0.433 |
| CSFV/AF326963 | 0.284 | 0.298 | 0.274 | 0.272 | 0.407 | 0.641 | 0.215 | | 0.278 | 0.505 | 0.433 |
| Aydin-like/JX428945 | 0.269 | 0.293 | 0.267 | 0.263 | 0.411 | 0.644 | 0.208 | 0.198 | | 0.504 | 0.437 |
| Rat/KJ950914 | 0.589 | 0.596 | 0.590 | 0.593 | 0.592 | 0.672 | 0.593 | 0.590 | 0.593 | | 0.503 |
| Bungowannah/EF100713 | 0.462 | 0.467 | 0.466 | 0.471 | 0.479 | 0.661 | 0.464 | 0.468 | 0.457 | 0.586 | |
